# Prevalence and risk factors of metabolic associated fatty liver disease in the contemporary South China population

**DOI:** 10.1186/s12986-021-00611-x

**Published:** 2021-09-08

**Authors:** Jiahua Fan, Shiyun Luo, Yongxin Ye, Jingmeng Ju, Zhuoyu Zhang, Ludi Liu, Jialu Yang, Min Xia

**Affiliations:** Guangdong Provincial Key Laboratory of Food, Nutrition and Health, Guangdong Engineering Technology Research Center of Nutrition Translation, Department of Nutrition, School of Public Health, Sun Yat-Sen University (Northern Campus), Guangzhou, Guangdong Province People’s Republic of China

**Keywords:** MAFLD, Prevalence, Fatty liver, Metabolic disorder

## Abstract

**Background:**

As a newly proposed diagnosis, data on the prevalence of metabolic dysfunction-associated fatty liver disease (MAFLD) is rare. We aimed to assess the prevalence and risk factors of MAFLD using new definition in the contemporary South China population.

**Methods:**

In this population based, cross sectional study, a total of 5377 participants aged 30–79 years old were recruited from the South China between 2018 and 2019. MAFLD was diagnosed in subjects who have both hepatic steatosis and metabolic disorders according to the newly international expert consensus. The total prevalence of MAFLD and prevalence by sex and age was estimated. Demographic characteristics, history of disease, and lifestyle were recorded by participants on a questionnaire. Abdominal ultrasonography was performed and evaluated by experienced sonographers. Multivariable logistic regression was used to calculate the odds ratios (ORs) of MAFLD.

**Results:**

Overall prevalence of MAFLD was 29.2% (95% confidence interval [CI] 28.0% to 30.5%). Prevalence was higher in women (31.7%) than in men (25.5%; *p* < 0.001 for sex difference) and in subjects aged 50 years or older (30.7%) than in those aged 30–49 years (19.8%; *p* < 0.001 for age difference). In participants diagnosed with MAFLD, the prevalence of overweight/obesity was up to 90.5%, type 2 diabetes (T2DM) and metabolic dysregulation were 25.0% and 62.2%, respectively. Risk factors for MAFLD included overweight/obesity (OR = 4.67; 95% CI, 3.76–5.83), T2DM (OR = 2.41, 95% CI, 1.68–3.47), hypertriglyceridemia (OR = 2.42, 95% CI, 2.03–2.87), high school education (OR = 1.50, 95% CI, 1.23–1.82), high income (OR = 1.22, 95% CI, 1.05–1.42). A lower risk of MAFLD was associated with high physical activity equivalent (OR = 0.71, 95% CI, 0.60–0.85). A U-shaped association of frequency of soups and ORs of MAFLD was found, the adjusted ORs (95% CI) of lower and higher frequency of soups were 1.58 (1.32–1.89) and 1.36 (1.13–1.63), respectively.

**Conclusions:**

Our results showed a high prevalence of MAFLD in the general adult population in South China. Obesity has the greatest impact on MAFLD, physical activity and moderate consumption of soups might be the potential protective factors of MAFLD.

**Supplementary Information:**

The online version contains supplementary material available at 10.1186/s12986-021-00611-x.

## Background

The rapid economic development in the last four decades has led to a large number of aging population and a change in lifestyle among the Chinese population, with a higher level of calorie intake and sedentary behavior, and lower level of physical activity [1–3]. This change has resulted in more people who are overweight/obesity, as well as metabolic diseases, including fatty liver disease (FLD), occurring [4].

Metabolic dysfunction-associated fatty liver disease (MAFLD), formerly named non-alcoholic fatty liver disease (NAFLD), is a newly proposed diagnosis revised by the international expert consensus, and has attracted substantial attention [5]. According to the consensus, MAFLD is diagnosed in subjects who have both hepatic steatosis and metabolic risk factors, including an elevated BMI (overweight/obesity), type 2 diabetes mellitus (T2DM) or at least two metabolic risk abnormalities. Besides, the definition of MAFLD is regardless of alcohol consumption or other concomitant liver diseases, which is different from the previous term NAFLD. Previous studies had reported that about one-third of adults in the general population affected by NAFLD and the prevalence of NAFLD increased significantly over time, which increased from 25.28 to 33.9% from 2005 to 2017 in China [6]. Progression of NAFLD can lead to fibrosis, cirrhosis, hepatocellular carcinoma and liver failure, as well as contribute to an increased risk for metabolic and cardiovascular morbidity and mortality, posing a major health and economic burden to all societies [7, 8]. Hence, NAFLD has become an important public health issue, prevention and treatment thereof are of strong public interest.

However, the most recent prevalence and risk factors of new definition MAFLD in China are rare. Only a small number of studies with different age, non-natural population or non-community population have been performed in Central and Southwest China [9–11]. Furthermore, as an important factor of MAFLD, lifestyle varies between different geographical distribution, especially dietary habits. For example, many communities have developed their own specific type of soup, such as Hot and Sour Soup in Southwest China, Mutton Soup in Northern China, and long-boiled soups in South China [12, 13]. However, no previous study had explored the relationship between the habit of eating soup and MAFLD. Therefore, the objective of this study was to estimate the prevalence and risk factors of MAFLD using new definition in the community population of South China.

## Methods

### Study population

This cross-sectional study participants were recruited from a community population who visited the Community Healthcare Center of Chashan Town in Dongguan, Guangdong province, which is one of the investigation points of South China Cohort project (SCC), from March 2018 to September 2019. Participants were informed of fasting 10 h before attending to the investigation site. A total of 5430 adults aged 30–79 years old with comprehensive anthropometric measurements, clinical examinations, abdominal ultrasound and questionnaire were included in the study. 53 subjects were excluded from the study, among them 41 subjects who lack data to define MAFLD, 4 subjects with history of liver cirrhosis, hepatectomy, schistosomal liver and liver cancer, and 8 subjects were excluded due to the extreme outlier of waist circumference (WC) and body mass index (BMI) according to three times standard deviation. Finally, 5377 participants with complete physical examination and laboratory test data were included in the final analysis (Additional file 1: Fig. S1).

All subjects of this study have signed informed consent and the study protocol was approved by the Ethics Committee of School of Public Health, Sun Yat-Sen University.

### Assessment of risk factors

Demographic characteristics, medical history, medication use and lifestyles were obtained by trained staff using a standard questionnaire. Current smoking was defined as consumption more than one cigarette per day over the past six months. Drinking was defined as drinking more than once per week over the past 1 year. Physical activity levels were assessed using the International Physical Activity Questionnaire Short Form (IPAQ-SF), and physical activity metabolic equivalents were calculated (MET-minutes per week). Long-boiled soups, the special diet habits of Guangdong, were asked as follows, “During the past 12 months, how often did you eat any long-boiled soups (never/occasionally, less than weekly, 1–2 per week, 3–4 per week, 5–6 per week, or daily)?”. Anthropometric parameters, including blood pressure, height, weight and WC, were measured using standardized procedures by trained examiners. Abdominal ultrasonography was performed and evaluated by experienced sonographer (Aplio400, TOSHIBA, Japan). Blood pressure (BP) was measured on the right upper arm in the sitting position after 10–15 min of rest using a validated digital automatic analyzer (Omron HEM-7136). Pulse pressure was calculated by subtracting the diastolic from the systolic blood pressure and used as a marker of arterial stiffness. Venous blood samples of all participants were collected after at least 10 h of fasting and were immediately processed and analyzed at the clinical laboratory of the Community Healthcare Center of Chashan Town (Dongguan) and performed with an automated analyzer (Mindray BS800M; Mindray, Shenzhen, China). Hypertension was defined as systolic blood pressure (SBP) ≥ 140 mmHg, diastolic blood pressure (DBP) ≥ 90 mmHg, or current use of antihypertensive medication [14].

### Diagnosis of MAFLD

The diagnosis of MAFLD was based on the ultrasonically diagnosed hepatic steatosis and the presence of one of the following three criteria [5]: overweight/obesity (defined as BMI ≥ 23 kg/m^2^), T2DM, or metabolic dysregulation. T2DM was defined based on self-reported history of diabetes determined previously by a healthcare professional, or fasting plasma glucose (FPG) ≥ 126 mg/dL (7.0 mmol/L). Metabolic dysregulation was defined by the presence of at least two of the following metabolic risk abnormalities: (a) WC ≥ 90/80 cm in men and women; (b) blood pressure ≥ 130/85 mmHg or specific drug treatment; (c) plasma triglycerides (TG) ≥ 1.70 mmol/L or specific drug treatment; (d) plasma HDL-cholesterol (HDL-C) < 1.0 mmol/L for men and < 1.3 mmol/L for women or specific drug treatment; (e) prediabetes (FPG levels 5.6 to 6.9 mmol/L). The detailed diagnosis was also presented in Additional file 1: Table S1.

### Statistical analysis

Characteristics of the study participants were explored according to the presence of MAFLD. Normally distributed continuous variables are reported as means ± SDs, and skewed variables are presented as medians (Interquartile ranges, IQR). Categorical variables are presented as a number (%). Independent-samples Student t test for normally distributed variables and Mann–Whitney U test for variables with highly skewed distributions. χ^2^ test was used to compare categorical variables. The Univariate logistic regression models with a cubic natural spline analysis were used to test the potential relationship between continuous variables and the odds ratios (ORs) of MAFLD. To evaluate the association of MAFLD across categories of risk factors, we divided the variables with statistical significance into category variables according to the clinical cut-off or the inflection point found by the cubic natural spline analysis. Among them, high physical activity was defined as physical activity metabolic equivalents in the highest quartile (physical activity metabolic equivalents ≥ 3465, MET-min/week). Logistic regression models ascertained the risk factors associated with MAFLD, including age, sex, education level, income, hypertension, diabetes, overweight, central obesity, metabolic dysregulation, antihypertensive, hypoglycemic drugs, lipid-lowering drugs, hypertriglyceridemia, HDL-C abnormality, ALT abnormality, hyperuricemia, physical activity metabolic equivalents and the frequency of long-boiled soups. These variables were mutually adjusted in the model.

All *p* values were two-tailed, and values of *p* < 0.05 were considered statistically significant. We used R version 4.04 (The R Foundation for Statistical Computing, Vienna, Austria) and SPSS 22.0 (IBM, Armonk, NY, USA) for data analysis.

## Results

### Baseline characteristics of participants with MAFLD in general participants

A total of 5377 participants recruited from Guangdong province, one of the investigation points of SCC (Additional file 1: Fig. S1). The median age of all the participants was 67 years (interquartile range: 60 to 71 years), with 2173 (40.4%) participants were men, and a mean BMI of 24.31 ± 3.52 kg/m^2^. T2DM and hypertension were identified in 830 (15.4%) and 3136 (58.3%) participants, respectively. MAFLD was diagnosed in 1571/5377 (29.2%) participants (Table 1). Compared to participants without MAFLD, patients with MAFLD were older, had a higher prevalence of overweight/obesity, central obesity, T2DM, hypertension and metabolic dysregulation, as well as a higher level of uric acid (UA) and higher proportion of drug usage. In addition, the frequency of soups and level of physical activity were significantly different between participants with or without MAFLD and a lower physical activity was found in patients with MAFLD (Table 1).

**Table 1:** Characteristics of participants according to the presence of MAFLD

Variables	Total (n = 5377)	Non-MAFLD (n = 3806)	MAFLD (n = 1571)	*p* value
*Demographic variables*				
Men (%)	2173 (40.4)	1618 (42.5)	555 (35.3)	< 0.001
Age (years)	67 (60–71)	67 (57–71)	67 (63–71)	< 0.001
Married (%)	4744 (88.4)	3363 (88.5)	1381 (88.2)	0.825
High school and above (%)	963 (17.9)	654 (17.2)	309 (19.7)	0.033
High-income (%)	2831 (53.5)	1931 (51.5)	900 (58.5)	< 0.001
*Chronic disease*				
Hypertension (%)	3136 (58.3)	2022 (53.1)	1114 (70.9)	< 0.001
T2DM (%)	830 (15.4)	438 (11.5)	392 (25.0)	< 0.001
Metabolic dysregulation (%)	1888 (35.2)	913 (24.1)	975 (62.2)	< 0.001
Overweight/obesity (%)	3419 (63.7)	2000 (52.7)	1419 (90.5)	< 0.001
Central obesity (%)	3001 (56.0)	1694 (44.6)	1307 (83.4)	< 0.001
*Drugs*				
Antihypertensive (%)	1771 (33.1)	1060 (28.0)	711 (45.6)	< 0.001
Hypoglycemic drugs (%)	628 (11.8)	335 (8.9)	293 (18.8)	< 0.001
Lipid-lowering drugs (%)	152 (2.8)	96 (2.5)	56 (3.6)	0.042
*Physical index*				
BMI (kg/m^2^)	24.31 ± 3.52	23.3 ± 3.12	26.75 ± 3.22	< 0.001
WC (cm)	85.48 ± 9.54	82.98 ± 8.87	91.53 ± 8.32	< 0.001
SBP (mmHg)	136.77 ± 19.50	135.10 ± 19.61	140.80 ± 18.65	< 0.001
DBP (mmHg)	81.52 ± 10.84	80.90 ± 10.89	83.01 ± 10.58	< 0.001
Pulse pressure (mmHg)	55.25 ± 15.36	54.20 ± 15.15	57.79 ± 15.58	< 0.001
MAP (mmHg)	99.94 ± 12.36	98.97 ± 12.5	102.28 ± 11.69	< 0.001
*Clinical index*				< 0.001
FPG (mmol/L)	4.95 (4.55–5.52)	4.85 (4.48–5.33)	5.27 (4.77–6.03)	< 0.001
ALT (IU/L)	20 (16–27)	19 (15–25)	23 (18–32)	< 0.001
AST (IU/L)	22 (18–26)	21 (18–25)	22 (19–27)	< 0.001
TG (mmol/L)	1.34 (0.96–1.95)	1.20 (0.88–1.69)	1.80 (1.29–2.53)	< 0.001
TC (mmol/L)	5.23 ± 1.07	5.2 ± 1.07	5.31 ± 1.07	< 0.001
HDL-C (mmol/L)	1.36 ± 0.34	1.4 ± 0.36	1.25 ± 0.27	< 0.001
LDL-C (mmol/L)	3.21 ± 0.91	3.19 ± 0.91	3.27 ± 0.92	0.003
UA (μmol/L)	364.68 ± 98.07	353.02 ± 95.35	392.92 ± 98.86	< 0.001
*Lifestyle*				
Current smoking (%)	724 (13.5)	584 (15.3)	140 (8.9)	< 0.001
Drinking (%)	267 (5.0)	182 (4.8)	85 (5.4)	0.366
Physical activity (MET-min/week)	1779 (1386–3465)	2076 (1386–3906)	1386 (1386–3066)	< 0.001
Frequency of soups (%)				< 0.001
< 1 per month	894 (16.8)	580 (15.4)	314 (20.2)	
< 1 per week	166 (3.1)	113 (3.0)	53 (3.4)	
1–2 per week	712 (13.4)	495 (13.1)	217 (14.0)	
3–4 per week	2083 (39.1)	1588 (42.0)	495 (31.9)	
5–6 per week	730 (13.7)	530 (14.0)	200 (12.9)	
Daily	745 (14.0)	471 (12.5)	274 (17.6)	

Values were means ± SD or medians in cases of continuous variables and numbers (percentages) in case of categorical data. For differences across the groups of calculated with χ^2^ test and analysis of variance for categorical and continuous data, respectively

*MAFLD* metabolic dysfunction-associated fatty liver disease, *T2DM* type 2 diabetes mellitus, *FPG* fasting plasma glucose, *LDL* low-density lipoprotein, *TG* triglyceride, *BMI* body mass index, *BP* blood pressure, *MAP* mean arterial pressure, *TC* total cholesterol, *HDL* high-density lipoprotein, *ALT* alanine aminotransferase, *AST* aspartate aminotransferase, *SD* standard deviation

### Prevalence of MAFLD and stratification by age, sex, conditions and BMI

**Fig. 1:**
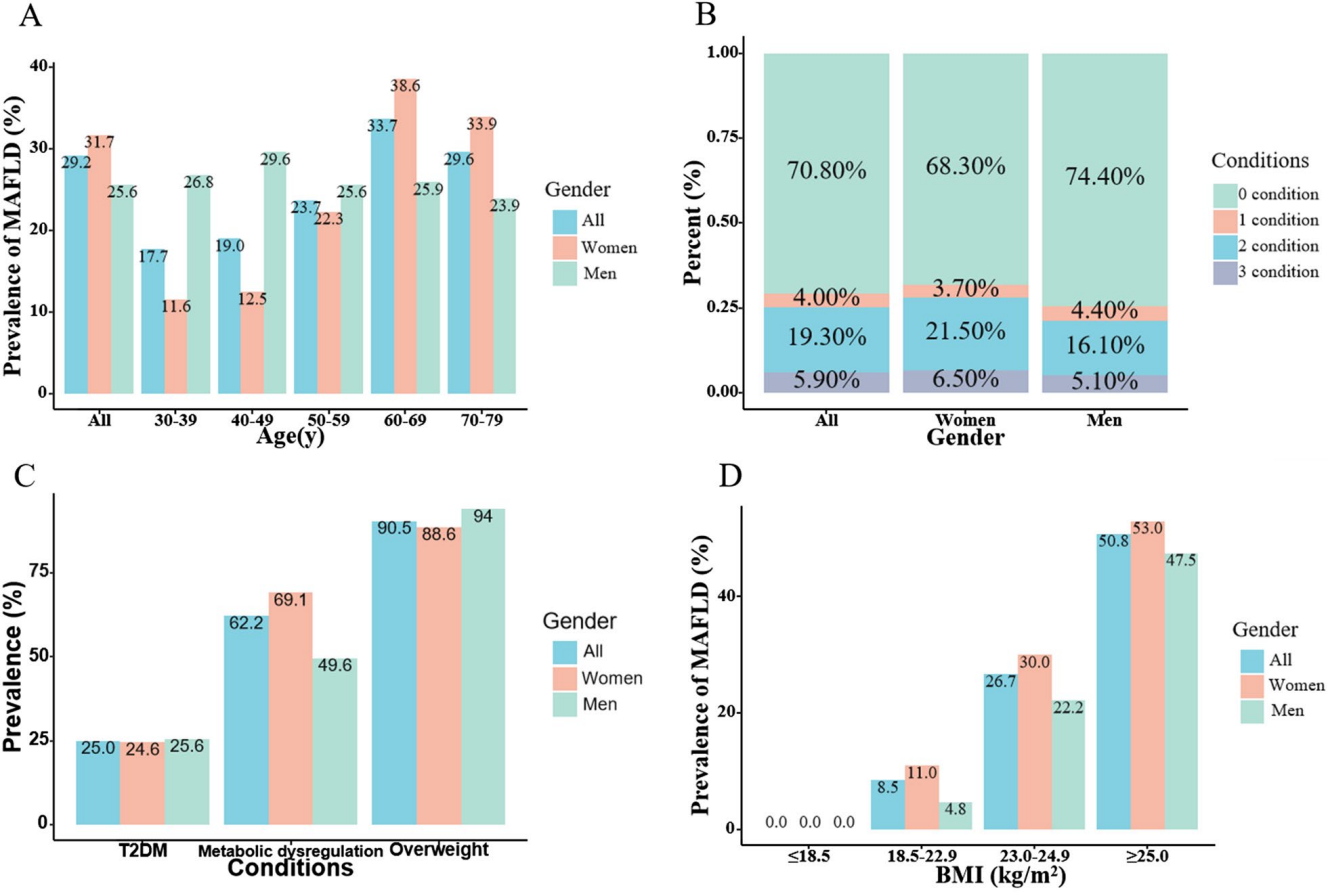
The prevalence of MAFLD according to age, sex, conditions and BMI. **A** The prevalence of MAFLD according to different age and gender. **B** The proportion of meeting one condition, two conditions or three conditions for MAFLD diagnosis according to gender. **C** The prevalence of each condition for diagnosis of MAFLD in participants with MALFD. **D** The prevalence of MAFLD according to different BMI levels

The prevalence of MAFLD was a significant difference between sex and the prevalence was higher in women than in men (31.7% vs. 25.5%, *p* < 0.001), the detailed differences between men and women were illustrated in Additional file 1: Table S2. The age-specific prevalence of MAFLD was shown in Fig. 1. For the total participants, the prevalence tended to rise with the increasing age, with a peak prevalence of 33.7% in the 60–69 age range. For women, the prevalence of MAFLD rose with age (*p* < 0.05), while there was no difference between different ages in men (*p* > 0.05). Besides, the proportion of patients who meet two or three conditions for MAFLD diagnosis was higher in women than in men (*p* < 0.05). Among patients with MAFLD, the prevalence of overweight was 90.5%, which is the highest among all the three conditions for MAFLD diagnosis, while T2DM and metabolic dysregulation were 25.0% and 62.2%, respectively. Consistently, women had a higher proportion of metabolic dysregulation than men.

### Relationship between risk factors and MAFLD

To test the potential relationship between continuous variables and the ORs of MAFLD, the univariate logistic regression models with a cubic natural spline analysis were performed. In all the participants, age, BMI, WC, SBP, DBP, TG, ALT, FPG and UA were found to be positively associated with ORs of MALFD, and there was a negative association of HDL and high level of physical activity with ORs of MALFD. The trend in women was consistent with the whole participants, while the age in men was not correlated with OR of MALFD (Additional file 1: Fig. S2). Most notably, there was a U-shaped relationship between frequency of soups and OR of MAFLD, with both lower (< 1 per week) and higher (≥ 3 per week) frequency of soups associated with higher ORs of MAFLD (Additional file 1: Fig. S2).

**Fig. 2:**
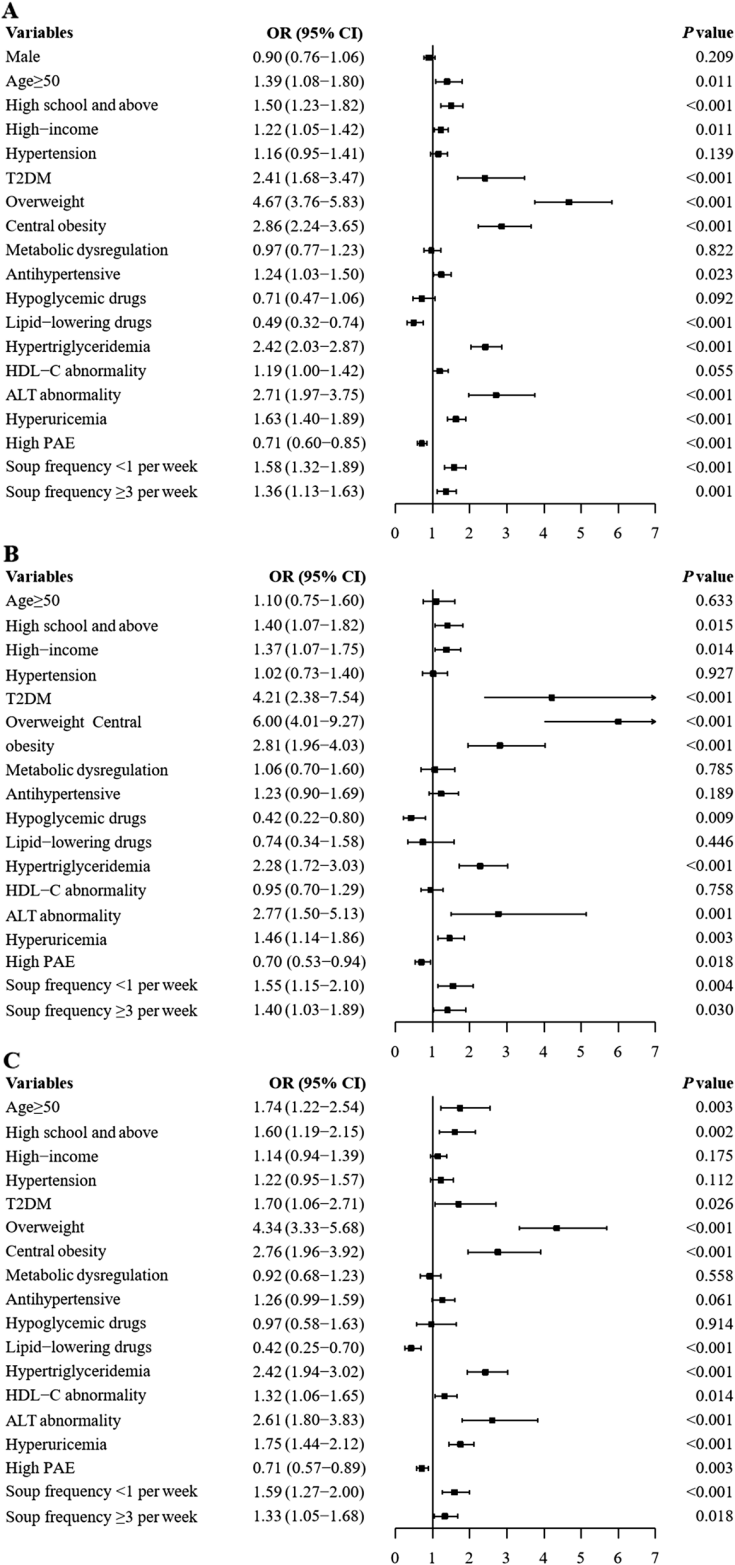
Multivariable logistic regression explored the risk factors of MAFLD. **A** All participants; **B** Men; **C** Women. Multivariable logistic regression adjustment for sex, age, hypertension, T2DM, overweight, central obesity, metabolic dysregulation, antihypertensive, hypoglycemic drugs, lipid-lowering drugs, hypertriglyceridemia, HDL-C abnormal, ALT abnormal, hyperuricemia, high level of physical activity and frequency of soups. *ORs* odds ratio, *PAE* physical activity equivalent

We further divided the continuous variables with statistical significance into category variables based on the clinical cut-off or the inflection point found by the cubic natural spline analysis to evaluate the association of MAFLD across categories of risk factors. The categories and the prevalence rates (%) 95% CI were presented in the Table 2, which showed significant differences of prevalence of MAFLD between groups. Multivariable logistic regression model adjustment for all the variables in Table 2 and results showed that age over 50 years old, high school and above, high family income, presence of T2DM, hypertension, overweight/obesity, central obesity, hypertriglyceridemia, ALT abnormal and hyperuricemia were risk factors of MAFLD, even after adjusting for confounding factors. While lipid-lowering drugs and high level of physical activity were protective factors of MAFLD. It is noteworthy that the U-shaped association of frequency of soups and ORs of MAFLD remained significant even after adjusted confounding factors, the adjusted ORs 95%CI of lower and higher frequency of soups were 1.58 (1.32–1.89) and 1.36 (1.13–1.63), respectively (Fig. 2).

**Table 2:** The prevalence of MAFLD in different subgroups

Variable	Category	Fraction	Prevalence rates (95% CI)	*p* value
Age (years)	< 50	145/731	19.8 (17.0–22.9)	< 0.001
	≥ 50	1426/4646	30.7 (29.4–32.0)	
Sex	Male	555/2173	25.5 (23.7–27.4)	< 0.001
	Female	1016/3204	31.7 (30.1–33.3)	
High school and above	No	1259/4407	28.6 (27.2–29.9)	0.030
	Yes	309/963	32.1 (29.1–35.1)	
High-income	No	638/2460	25.9 (24.2–27.7)	< 0.001
	Yes	900/2831	31.8 (30.1–33.5)	
Menopause	No	83/514	16.1 (13.1–19.6)	< 0.001
	Yes	932/2689	34.7 (32.9–36.5)	
Hypertension	No	457/2241	20.4 (18.7–22.1)	< 0.001
	Yes	1114/3136	35.5 (33.8–37.2)	
T2DM	No	1179/4547	25.9 (24.7–27.2)	< 0.001
	Yes	392/830	47.2 (43.8–50.7)	
Metabolic dysregulation	No	592/3475	17.0 (15.8–18.3)	< 0.001
	Yes	975/1888	51.6 (49.4–53.9)	
Overweight/obesity	No	149/1945	7.7 (6.5–8.9)	< 0.001
	Yes	1419/3419	41.5 (39.8–43.2)	
Central obesity	No	260/2362	11.0 (9.7–12.3)	< 0.001
	Yes	1307/3001	43.6 (41.8–45.3)	
ALT abnormality	No	1406/5063	27.8 (26.5–29.0)	< 0.001
	Yes	151/264	57.2 (51.0–63.2)	
Hypertriglyceridemia	No	690/3463	19.9 (18.6–21.3)	< 0.001
	Yes	867/1864	46.5 (44.2–48.8)	
HDL-C abnormality	No	824/3542	23.3 (21.9–24.7)	< 0.001
	Yes	733/1785	41.1 (38.8–43.4)	
Hyperuricemia	No	738/3349	22.0 (20.6–23.5)	< 0.001
	Yes	819/1978	41.4 (39.2–43.6)	
High physical activity	No	1178/3878	30.4 (28.9–31.9)	0.002
	Yes	386/1484	26.0 (23.8–28.3)	
Frequency of soups	< 1 per week	584/1772	33.0 (30.8–35.2)	< 0.001
	≥ 3 per week	474/1475	32.1 (29.8–34.6)	

*MAFLD* metabolic dysfunction-associated fatty liver disease, *T2DM* type 2 diabetes mellitus, *HDL-C* high-density lipoprotein cholesterol, *ALT* alanine aminotransferase, *AST* aspartate aminotransferase

### Joint association between frequency of soups and physical activity equivalent

**Fig. 3:**
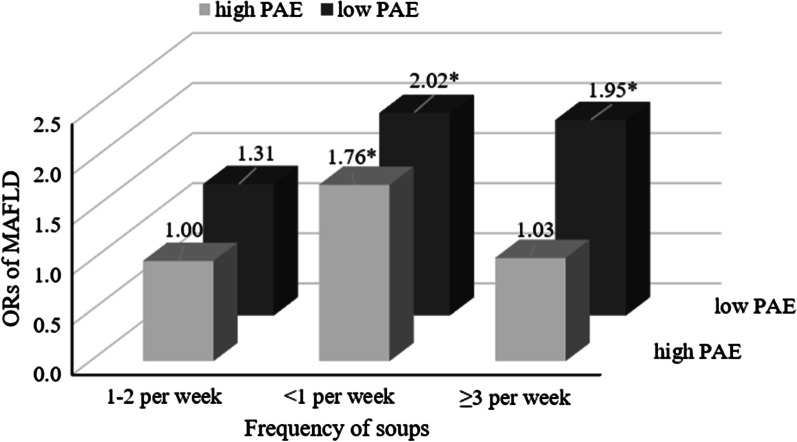
Multivariable-adjusted ORs for MAFLD for joint association between frequency of soups and level of physical activity. The multivariable logistic regression model was adjusted for sex, age, hypertension, T2DM, overweight, central obesity, metabolic dysregulation, antihypertensive, hypoglycemic drugs, lipid-lowering drugs, hypertriglyceridemia, HDL-C abnormal, ALT abnormal, hyperuricemia, high level of physical activity and frequency of soups. The *p* for interaction is < 0.001. *p value < 0.05. *ORs* odds ratio, *PAE* physical activity equivalent

Moreover, interaction analysis showed that an interaction between level of physical activity and frequency of soups (*p* < 0.05). We further used joint analysis to explore the influence of lifestyle (physical activity and frequency of soups) in MAFLD (Fig. 3). Participants were cross-classified on the basis of level of physical activity and frequency of soups. 20.3% participants with high level of physical activity and consumption of soups for 1–2 per week were diagnosed with MAFLD, which is the lowest prevalence in all the groups. We then used participants with high level of physical activity and frequency of soups 1–2 per week group as reference, multivariable-adjusted logistic regression showed that participants with lower frequency of soups had higher ORs of MAFLD, regardless of level of physical activity level. Participants with a low level of physical activity and higher frequency of soups also had a significant association with ORs of MAFLD, while there was no relationship between high level of physical activity and high frequency of soups group (*p* < 0.05).

### Sensitivity analyses

We also performed sensitivity analyses in this study. In sensitivity analysis, we examined the effect of age and sex on the robustness association between risk factors and MAFLD. All the significant associations from the sensitivity analysis were consistent with the primary analyses, except the Lipid-lowering drugs (Additional file 1: Table S3).

## Discussion

In this cross-sectional study, the prevalence and risk factors for MAFLD were explored, and significant differences in the prevalence of MAFLD among groups based on sex, age, BMI, and life style were revealed. To our knowledge, this study is the first to explore the prevalence and risk factors of MAFLD in the community population of South China since the new definition of MAFLD was established.

Our study found that the overall prevalence of MAFLD was 29.2% and the prevalence rose with age, which was consistently with the prevalence of 31.38% found in the rural population of Xinxiang, Henan province [9], but higher than the prevalence reported by the study conducted in a health examination population [10]. In addition, previous studies reported a higher prevalence of MAFLD in men than in women, while our study showed an opposite result. Possible reasons for the inconsistent results might be due to different age of study population. The median age of our study population was 67 years old, which was older than previous studies. More aged means a higher proportion of postmenopausal, while menopause was a risk factor for MAFLD. This was also supported by our study which found that female participants over 50 years old, the OR of MAFLD was 1.74-times higher than that of under 50 years old.

Overweight and obesity play a crucial role in the development of FLD [4, 15, 16]. Individuals with obesity show characteristic imbalance of metabolic profile which is associated with profound changes in insulin sensitivity, inflammatory reaction and other biochemical metabolites alterations, making an individual more potential to metabolic disorders [17]. In fact, previous studies had reported that FLD exhibits a high degree of comorbidity with disorders of the metabolic syndrome and they share the same pathogenic mechanisms of disease [18]. In our study, we also found that the prevalence of MAFLD increased with the increase of BMI and overweight/obesity accounts for 90.5% of MAFLD. Multivariable logistic regression revealed participants with overweight/obesity had 4.67-times ORs of MAFLD than lean participants, indicating a main influence of overweight/obesity in MAFLD. While for those non-obese participants with MAFLD, diagnosis by the presence of other metabolic abnormalities, including lipids and glucose levels, the mechanisms need to be examined in more future studies.

Changing lifestyles have contributed to the obesity and FLD epidemic [6, 19, 20]. Physical activity has been well-recognized to reduce the incidence and mortality of various chronic diseases, including cardiovascular disease, diabetes, stroke and several types of cancer [21–23]. Previous studies had reported an inverse linear association between physical activity and the prevalence of NAFLD [24–26]. Whether the association between physical activity and the new definition of MAFLD was the same as previous studies remained unclear. In this study, we observed that participants with MAFLD had a lower level of physical activity and multivariable logistic regression revealed that a higher level of physical activity was associated with a lower OR of MAFLD, which OR of MAFLD was 29% lower among subjects with higher level of physical activity. This finding was consistent with earlier studies. For the relationship between diet and MAFLD, we explored the frequency of long-boiled soups, which is the special diet habits of Guangdong, South China. Previous long-term studies have found a beneficial effect of soup consumption on body weight [27] or metabolic profiles [28, 29], which possibly by delaying gastric emptying and increasing glycemic response, thus reducing appetite or energy intake [30–32]. Our study also found that consumption of soups was associated with MAFLD, logistic regression models with a cubic natural spline analysis showed that there was a U-shaped association of frequency of soups and ORs of MAFLD even after adjustment for confounding factors, with both lower (< 1 per week) and higher (≥ 3 per week) frequency of soups associated with higher ORs of MAFLD. Our study was consistent with a previous study and further proved the relationship between soups and MAFLD. Nevertheless, the exact molecular mechanisms driving this phenomenon need to be examined in future studies.

There were several limitations to our study. Firstly, this was a cross-sectional study, which does not allow for establishing the temporality and causality of the observed associations, as well as the influence of lifestyle change on MAFLD. Secondly, we do not detect the level of insulin resistance and C-reactive protein, which are the components of metabolic disorders for the definition of MAFLD. However, as all the participants diagnosis of hepatic steatosis were included in the MAFLD group based on the new definition of MAFLD, the overall prevalence of MAFLD was not affected. Finally, as our participants were all recruited from South China, our findings might not be necessarily generalizable to populations from other regions, which should be studied in the future.

## Conclusion

In conclusion, this study showed a high prevalence of MAFLD in the South China population. The prevalence of MAFLD in the study population was 29.2%. Among all the risk factors, obesity had the greatest impact on MAFLD, while physical activity and moderate consumption of soups might be the potential protective factors of MAFLD.

## Supplementary Information

**Additional file 1: Fig. S1.** Flow chart of the study population. **Table S1.** Diagnostic criteria for MAFLD in the present study. **Table S2.** Characteristics of participants according to gender. **Fig. S2.** The relationship between continuous variables and ORs of MAFLD. **Table S3.** ORs for MAFLD after adjustment for age and sex.

## Data Availability

The datasets in the current study are available from the corresponding author on reasonable request.

## Abbreviations

FLD: Fatty liver disease
MAFLD: Metabolic dysfunction-associated fatty liver disease
T2DM: Type 2 diabetes mellitus
FPG: Fasting plasma glucose
LDL: Low-density lipoprotein
TG: Triglyceride
BMI: Body mass index
BP: Blood pressure
TC: Total cholesterol
HDL: High-density lipoprotein
ALT: Alanine aminotransferase
AST: Aspartate aminotransferase
SD: Standard deviation
IQR: Interquartile range
ORs: Odds ratios
95% CI: 95% Confidence interval

## References

[1] Inoue Y, Qin B, Poti J, Sokol R, Gordon-Larsen P (2018). Epidemiology of obesity in adults: latest trends. Curr Obes Rep.

[2] Popkin BM (2014). Synthesis and implications: China's nutrition transition in the context of changes across other low- and middle-income countries. Obes Rev.

[3] Du SF, Wang HJ, Zhang B, Zhai FY, Popkin BM (2014). China in the period of transition from scarcity and extensive undernutrition to emerging nutrition-related non-communicable diseases, 1949–1992. Obes Rev.

[4] Pan X-F, Wang L, Pan A (2021). Epidemiology and determinants of obesity in China. Lancet Diabetes Endocrinol.

[5] Eslam M, Newsome PN, Sarin SK, Anstee QM, Targher G, Romero-Gomez M, Zelber-Sagi S, Wai-Sun Wong V, Dufour JF, Schattenberg JM (2020). A new definition for metabolic dysfunction-associated fatty liver disease: an international expert consensus statement. J Hepatol.

[6] Li J, Zou B, Yeo YH, Feng Y, Xie X, Lee DH, Fujii H, Wu Y, Kam LY, Ji F (2019). Prevalence, incidence, and outcome of non-alcoholic fatty liver disease in Asia, 1999–2019: a systematic review and meta-analysis. Lancet Gastroenterol Hepatol.

[7] Alferink LJ, Kiefte-de Jong JC, Erler NS, Veldt BJ, Schoufour JD, de Knegt RJ, Ikram MA, Metselaar HJ, Janssen H, Franco OH, Darwish Murad S (2019). Association of dietary macronutrient composition and non-alcoholic fatty liver disease in an ageing population: the Rotterdam Study. Gut.

[8] Younossi Z, Tacke F, Arrese M, Chander Sharma B, Mostafa I, Bugianesi E, Wai-Sun Wong V, Yilmaz Y, George J, Fan J, Vos MB (2019). Global perspectives on nonalcoholic fatty liver disease and nonalcoholic steatohepatitis. Hepatology.

[9] Li H, Guo M, An Z, Meng J, Jiang J, Song J, Wu W (2020). Prevalence and risk factors of metabolic associated fatty liver disease in Xinxiang, China. Int J Environ Res Public Health.

[10] Chen YL, Li H, Li S, Xu Z, Tian S, Wu J, Liang XY, Li X, Liu ZL, Xiao J (2021). Prevalence of and risk factors for metabolic associated fatty liver disease in an urban population in China: a cross-sectional comparative study. BMC Gastroenterol.

[11] Liu D, Shen Y, Zhang R, Xun J, Wang J, Liu L, Steinhart C, Chen J, Lu H (2021). Prevalence and risk factors of metabolic associated fatty liver disease among people living with HIV in China. J Gastroenterol Hepatol.

[12] Liu Y, Liu Q, Li P, Xing D, Hu H, Li L, Hu X, Long C (2018). Plants traditionally used to make Cantonese slow-cooked soup in China. J Ethnobiol Ethnomed.

[13] Song F, Cho MS (2017). Geography of food consumption patterns between South and North China. Foods.

[14] Lu J, Lu Y, Wang X, Li X, Linderman GC, Wu C, Cheng X, Mu L, Zhang H, Liu J (2017). Prevalence, awareness, treatment, and control of hypertension in China: data from 1·7 million adults in a population-based screening study (China PEACE Million Persons Project). Lancet.

[15] Kwon YM, Oh SW, Hwang SS, Lee C, Kwon H, Chung GE (2012). Association of nonalcoholic fatty liver disease with components of metabolic syndrome according to body mass index in Korean adults. Am J Gastroenterol.

[16] Pang Q, Zhang JY, Song SD, Qu K, Xu XS, Liu SS, Liu C (2015). Central obesity and nonalcoholic fatty liver disease risk after adjusting for body mass index. World J Gastroenterol.

[17] Fan J, Liu Y, Yin S, Chen N, Bai X, Ke Q, Shen J, Xia M (2019). Small dense LDL cholesterol is associated with metabolic syndrome traits independently of obesity and inflammation. Nutr Metab (Lond).

[18] Sookoian S, Pirola CJ (2019). Review article: shared disease mechanisms between non-alcoholic fatty liver disease and metabolic syndrome—translating knowledge from systems biology to the bedside. Aliment Pharmacol Ther.

[19] Mahady SE, George J (2018). Predicting the future burden of NAFLD and NASH. J Hepatol.

[20] Estes C, Anstee QM, Arias-Loste MT, Bantel H, Bellentani S, Caballeria J, Colombo M, Craxi A, Crespo J, Day CP (2018). Modeling NAFLD disease burden in China, France, Germany, Italy, Japan, Spain, United Kingdom, and United States for the period 2016–2030. J Hepatol.

[21] Krishnan S, Rosenberg L, Palmer JR (2009). Physical activity and television watching in relation to risk of type 2 diabetes: the Black Women's Health Study. Am J Epidemiol.

[22] Sattelmair J, Pertman J, Ding EL, Kohl HW, Haskell W, Lee IM (2011). Dose response between physical activity and risk of coronary heart disease: a meta-analysis. Circulation.

[23] Manson JE, Greenland P, LaCroix AZ, Stefanick ML, Mouton CP, Oberman A, Perri MG, Sheps DS, Pettinger MB, Siscovick DS (2002). Walking compared with vigorous exercise for the prevention of cardiovascular events in women. N Engl J Med.

[24] Ryu S, Chang Y, Jung HS, Yun KE, Kwon MJ, Choi Y, Kim CW, Cho J, Suh BS, Cho YK (2015). Relationship of sitting time and physical activity with non-alcoholic fatty liver disease. J Hepatol.

[25] Hallsworth K, Thoma C, Moore S, Ploetz T, Anstee QM, Taylor R, Day CP, Trenell MI (2015). Non-alcoholic fatty liver disease is associated with higher levels of objectively measured sedentary behaviour and lower levels of physical activity than matched healthy controls. Frontline Gastroenterol.

[26] Gerber L, Otgonsuren M, Mishra A, Escheik C, Birerdinc A, Stepanova M, Younossi ZM (2012). Non-alcoholic fatty liver disease (NAFLD) is associated with low level of physical activity: a population-based study. Aliment Pharmacol Ther.

[27] Kuroda M, Ninomiya K (2020). Association between soup consumption and obesity: a systematic review with meta-analysis. Physiol Behav.

[28] Martínez-Tomás R, Larqué E, González-Silvera D, Sánchez-Campillo M, Burgos MI, Wellner A, Parra S, Bialek L, Alminger M, Pérez-Llamas F (2012). Effect of the consumption of a fruit and vegetable soup with high in vitro carotenoid bioaccessibility on serum carotenoid concentrations and markers of oxidative stress in young men. Eur J Nutr.

[29] Cugnet-Anceau C, Nazare JA, Biorklund M, Le Coquil E, Sassolas A, Sothier M, Holm J, Landin-Olsson M, Onning G, Laville M, Moulin P (2010). A controlled study of consumption of beta-glucan-enriched soups for 2 months by type 2 diabetic free-living subjects. Br J Nutr.

[30] Mattes R (2005). Soup and satiety. Physiol Behav.

[31] Zhu Y, Hsu WH, Hollis JH (2013). The effect of food form on satiety. Int J Food Sci Nutr.

[32] Clegg ME, Ranawana V, Shafat A, Henry CJ (2013). Soups increase satiety through delayed gastric emptying yet increased glycaemic response. Eur J Clin Nutr.

