# Supplementary material for: Prevalence and risk factors of metabolic associated fatty liver disease in the contemporary South China population
Source: Nutr Metab (Lond). 2021 Sep 8;18:82. doi: 10.1186/s12986-021-00611-x (PMC8425111; doi:10.1186/s12986-021-00611-x)
Supplement: Supplementary file 1 — Additional file 1: Fig. S1. Flow chart of the study population. Table S1. Diagnostic criteria for MAFLD in the present study. Table S2. Characteristics of participants according to gender. Fig. S2. The relationship between continuous variables and ORs of MAFLD. Table S3. ORs for MAFLD after adjustment for age and sex. [file 12986_2021_611_MOESM1_ESM.doc]

**Prevalence and Risk factors metabolic associated fatty liver disease in the contemporary South China population**

Abbreviated title: Prevalence of MAFLD

Jiahua Fan^1*^, Shiyun Luo^1*^, Yongxin Ye^1^, Jingmeng Ju^1^, Zhuoyu Zhang^1^, Ludi Liu^1^, Jialu Yang^1^, Min Xia^1#^

^1^ Guangdong Provincial Key Laboratory of Food, Nutrition and Health; Guangdong Engineering Technology Research Center of Nutrition Translation; Department of Nutrition, School of Public Health, Sun Yat-sen University (Northern Campus), Guangzhou, Guangdong Province, P.R. China.

* These Authors contributed equally to the work.

^#^ Corresponding author

Address all correspondence and requests for reprints to: Min Xia, Department of Nutrition, School of Public Health, Sun Yat-sen University (Northern Campus), Guangzhou, Guangdong Province, China. E-mail: [xiamin@mail.sysu.edu.cn](mailto:xiamin@mail.sysu.edu.cn)

Authors email addresses: Jiahua Fan^1*^, [fanjh23@mail.sysu.edu.cn](mailto:fanjh23@mail.sysu.edu.cn); Shiyun Luo ^1*^, luoshy25@mail2.sysu.edu.cn; Yongxin Ye ^1^, yeyx9@mail2.sysu.edu.cn; Jingmeng Ju ^1^, jujingm@mail2.sysu.edu.cn; Zhuoyu Zhang ^1^, zhangzhy226@mail2.sysu.edu.cn; Ludi Liu ^1^, liuld7@mail2.sysu.edu.cn; Jialu Yang ^1^, yangjlu@mail2.sysu.edu.cn; Min Xia^1#^, [xiamin@mail.sysu.edu.cn](mailto:xiamin@mail.sysu.edu.cn)


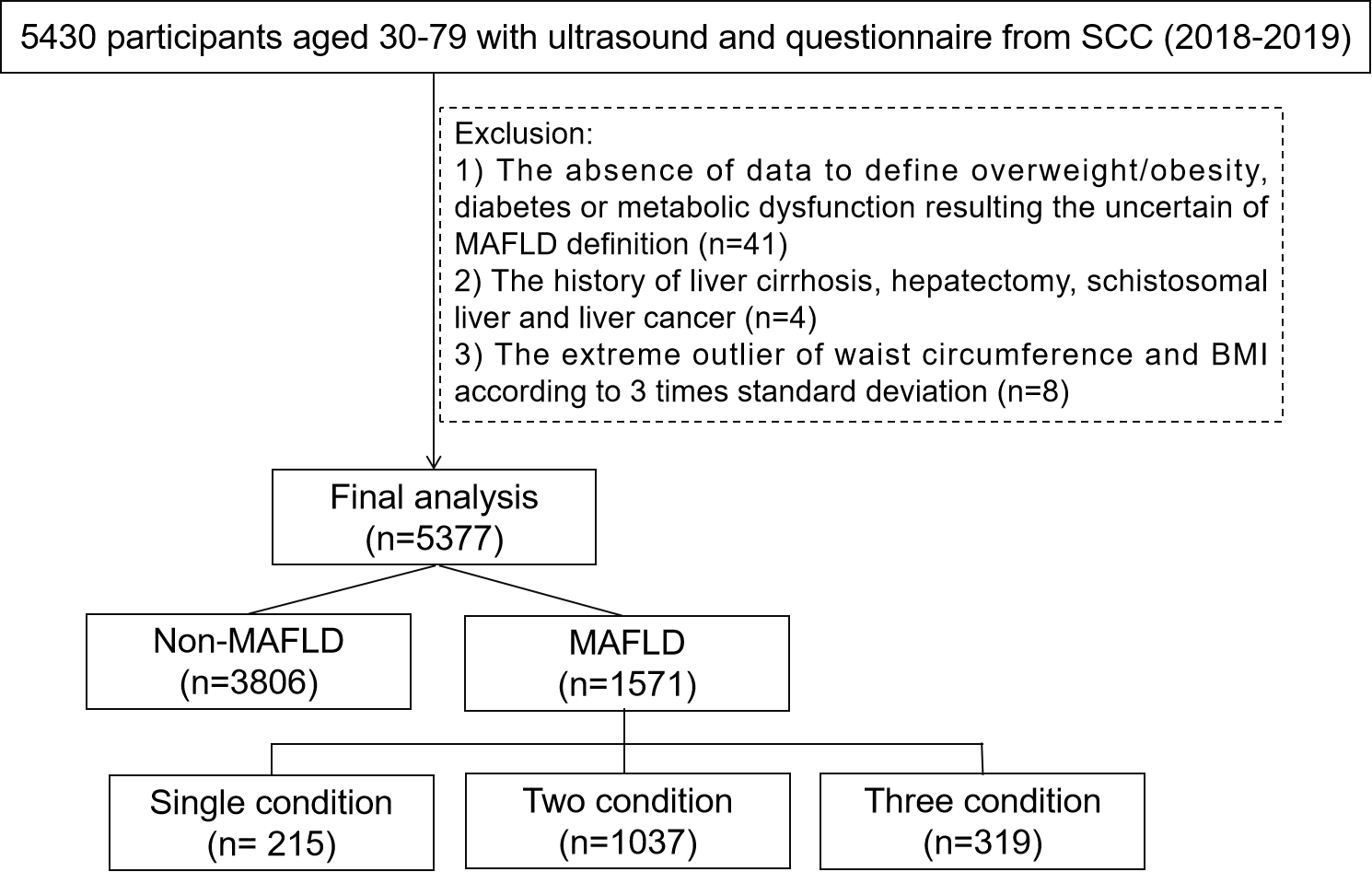


**Fig. S1 Flow chart of the study population**

Table S1 Diagnostic criteria for MAFLD in the present study

| **Participants with hepatic steatosis detected by B ultrasonography in addition to one of the following three criteria** | |
| --- | --- |
| Overweight/obesity | BMI ≥23 kg/m^2^ |
| Presence of type 2 diabetes mellitus (T2DM) | A self-reported diagnosis that was determined previously by a healthcare professional, or participants fasting plasma glucose ≥126 mg/dL (7.0 mmol/L) |
| Metabolic dysregulation | Presence of at least two metabolic risk abnormalities:  a. Waist circumference ≥90/80 cm in men and women;  b. Blood pressure ≥130/85 mmHg or specific drug treatment;  c. Plasma triglycerides ≥150 mg/dl (≥1.70 mmol/L) or specific drug treatment;  d. Plasma HDL-cholesterol <40 mg/dl (<1.0 mmol/L) for men and <50 mg/dl (<1.3 mmol/L) for women or specific drug treatment;  e. Prediabetes (fasting glucose levels 100-125 mg/dl [5.6-6.9 mmol/L]). |

Table S2 Characteristics of participants according to gender

| **Variables** | **Total**  **(n = 5377)** | **Women**  **(n = 3204)** | **Men**  **(n = 2173)** | ***P* value** |
| --- | --- | --- | --- | --- |
| Age (y) | 67 (60-71) | 67 (60-71) | 67 (60-71) | 0.023 |
| Married (%) | 4744 (88.4) | 2683 (84.0) | 2061 (94.9) | <0.001 |
| High school and above (%) | 963 (17.9) | 397 (12.4) | 566 (26.1) | <0.001 |
| High-income (%) | 2831 (53.5) | 1683 (53.4) | 1148 (53.6) | 0.890 |
| **Chronic disease** |  |  |  |  |
| Hypertension (%) | 3136 (58.3) | 1860 (58.1) | 1276 (58.7) | 0.646 |
| T2DM (%) | 830 (15.4) | 492 (15.4) | 338 (15.6) | 0.873 |
| Metabolic dysregulation (%) | 1888 (35.2) | 1377 (43.1) | 511 (23.6) | <0.001 |
| Overweight/obesity (%) | 3419 (63.7) | 2022 (63.3) | 1397 (64.4) | 0.397 |
| Central obesity (%) | 3001 (56.0) | 2197 (68.8) | 804 (37.1) | <0.001 |
| **Drugs** |  |  |  |  |
| Antihypertensive (%) | 1771 (33.1) | 1046 (32.8) | 725 (33.6) | 0.583 |
| Hypoglycemic drugs (%) | 628 (11.8) | 378 (11.9) | 250 (11.6) | 0.804 |
| Lipid-lowering drugs (%) | 152 (2.8) | 98 (3.1) | 54 (2.5) | 0.250 |
| **Physical** **index** |  |  |  |  |
| BMI (kg/m^2^) | 24.31 ± 3.52 | 24.37 ± 3.67 | 24.21 ± 3.28 | 0.097 |
| WC (cm) | 85.48 ± 9.54 | 84.65 ± 9.73 | 86.7 ± 9.12 | < 0.001 |
| SBP (mmHg) | 136.77 ± 19.5 | 137.24 ± 20.46 | 136.07 ± 17.98 | 0.027 |
| DBP (mmHg) | 81.52 ± 10.84 | 80.73 ± 10.7 | 82.68 ± 10.93 | < 0.001 |
| Pulse Pressure (mmHg) | 55.25 ± 15.36 | 56.51 ± 16.08 | 53.39 ± 14.03 | < 0.001 |
| MAP (mmHg) | 99.94 ± 12.36 | 99.57 ± 12.59 | 100.48 ± 11.98 | 0.008 |
| **Clinical index** |  |  |  |  |
| FPG (mmol/L) | 4.95 (4.55-5.52) | 4.94 (4.55-5.5) | 4.96 (4.54-5.53) | 0.602 |
| ALT (IU/L) | 20 (16-27) | 19 (15-25) | 21 (17-29) | < 0.001 |
| AST (IU/L) | 22 (18-26) | 21 (18-25) | 22 (19-26) | < 0.001 |
| TG (mmol/L) | 1.34 (0.96-1.95) | 1.37 (0.98-2) | 1.31 (0.93-1.89) | < 0.001 |
| TC (mmol/L) | 5.23 ± 1.07 | 5.38 ± 1.07 | 5.02 ± 1.03 | < 0.001 |
| HDL-C (mmol/L) | 1.36 ± 0.34 | 1.43 ± 0.34 | 1.25 ± 0.32 | < 0.001 |
| LDL-C (mmol/L) | 3.21 ± 0.91 | 3.27 ± 0.92 | 3.13 ± 0.89 | < 0.001 |
| UA (μmol/L) | 364.68 ± 98.07 | 339.98 ± 90.9 | 401.13 ± 96.91 | < 0.001 |
| **Lifestyle** |  |  |  |  |
| Current smoking (%) | 724 (13.5) | 7 (0.2) | 717 (33.0) | <0.001 |
| Drinking (%) | 267 (5.0) | 27 (0.8) | 240 (11.1) | <0.001 |
| Physical activity (MET-min/week) | 1779 (1386-3465) | 1908 (1386-3612) | 1680 (1386-3360) | 0.189 |
| MAFLD (%) | 1571 (29.2) | 1016 (31.7) | 555 (25.5) | <0.001 |
| MAFLD components (%) |  |  |  | <0.001 |
| Non-condition | 3806 (70.8) | 2188 (68.3) | 1618 (74.5) |  |
| One condition | 215 (4.0) | 120 (3.7) | 95 (4.4) |  |
| Two condition | 1037 (19.3) | 688 (21.5) | 349 (16.1) |  |
| Three condition | 319 (5.9) | 208 (6.5) | 111 (5.1) |  |

**
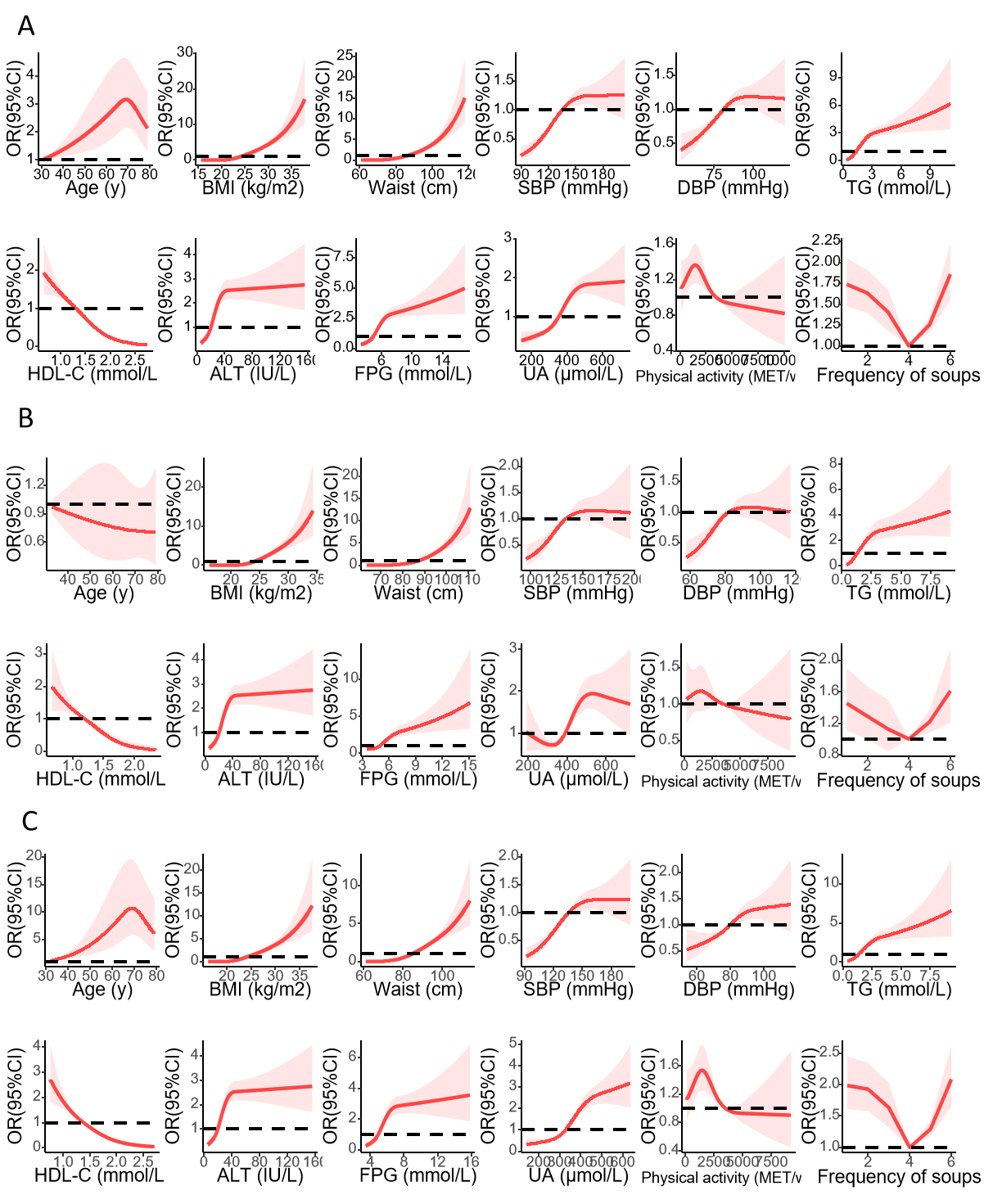
**

**Fig. S2 The relationship between continuous variables and ORs of MAFLD.** The univariate logistic regression models with a cubic natural spline analysis was performed. A, All participants; B, Men; C, Women. ORs=odds ratios.

**Table S3 ORs for MAFLD after adjustment for age and sex**

|  | **All (n=5377)** | | **Men (n=2173)** | | **Women (n=3204)** | |
| --- | --- | --- | --- | --- | --- | --- |
|  | **OR (95% CI)** | ***p* value** | **OR (95% CI)** | ***p* value** | **OR (95% CI)** | ***p* value** |
| High school and above | 1.37 (1.17-1.60) | <0.001 | 1.48 (1.19-1.82) | <0.001 | 1.33 (1.05-1.68) | 0.0163 |
| High-income | 1.28 (1.14-1.45) | <0.001 | 1.48 (1.21-1.81) | <0.001 | 1.17 (1.00-1.36) | 0.0507 |
| Antihypertensive | 2.04 (1.80-2.31) | <0.001 | 2.02 (1.65-2.47) | <0.001 | 2.02 (1.72-2.36) | <0.001 |
| Hypoglycemic drugs | 2.26 (1.91-2.68) | <0.001 | 2.17 (1.64-2.86) | <0.001 | 2.30 (1.84-2.86) | <0.001 |
| Lipid-lowering drugs | 1.33 (0.95-1.86) | 0.0954 | 1.76 (0.99-3.06) | 0.0474 | 1.11 (0.72-1.68) | 0.626 |
| Hypertension | 2.03 (1.79-2.31) | <0.001 | 1.98 (1.61-2.44) | <0.001 | 1.99 (1.69-2.35) | <0.001 |
| T2DM | 2.44 (2.09-2.85) | <0.001 | 2.56 (2.00-3.26) | <0.001 | 2.34 (1.92-2.85) | <0.001 |
| Overweight | 8.64 (7.24-10.4) | <0.001 | 13.27 (9.35-19.47) | <0.001 | 7.13 (5.79-8.85) | <0.001 |
| Central obesity | 6.70 (5.74-7.85) | <0.001 | 6.19 (5.02-7.66) | <0.001 | 6.94 (5.50-8.85) | <0.001 |
| Metabolic dysregulation | 5.09 (4.47-5.80) | <0.001 | 5.76 (4.65-7.17) | <0.001 | 4.50 (3.82-5.31) | <0.001 |
| Hypertriglyceridemia | 3.41 (3.01-3.86) | <0.001 | 3.47 (2.83-4.25) | <0.001 | 3.19 (2.72-3.74) | <0.001 |
| HDL-C abnormality | 2.19 (1.94-2.49) | <0.001 | 2.00 (1.61-2.48) | <0.001 | 2.24 (1.92-2.61) | <0.001 |
| ALT abnormality | 3.57 (2.77-4.63) | <0.001 | 2.56 (1.56-4.16) | <0.001 | 3.65 (2.69-4.97) | <0.001 |
| High-UA | 2.48 (2.20-2.81) | <0.001 | 1.95 (1.60-2.38) | <0.001 | 2.78 (2.38-3.26) | <0.001 |
| High physical activity | 0.68 (0.59-0.79) | <0.001 | 0.73 (0.57-0.93) | 0.0112 | 0.66 (0.55-0.80) | <0.001 |
| Soup frequency <1 per week | 1.49 (1.29-1.72) | <0.001 | 1.37 (1.08-1.74) | 0.00849 | 1.56 (1.30-1.87) | <0.001 |
| Soup frequency ≥3 per week | 1.47 (1.27-1.71) | <0.001 | 1.50 (1.17-1.91) | 0.00116 | 1.44 (1.19-1.74) | <0.001 |

ORs=odds ratios.
